# A comparative study of electropolymerization and photopolymerization for the determination of molnupiravir and their application in an electrochemical sensor via computationally designed molecularly imprinted polymers

**DOI:** 10.1007/s00604-024-06353-w

**Published:** 2024-04-17

**Authors:** Ahmet Cetinkaya, M. Altay Unal, Hasan Nazır, M. Emin Çorman, Lokman Uzun, Sibel A. Ozkan

**Affiliations:** 1 Faculty of Pharmacy, Department of Analytical Chemistry, Ankara University, Ankara, Turkey; 2 Graduate School of Health Sciences, Ankara University, Ankara, Turkey; 3 Stem Cell Institute, Ankara University, Ankara, Turkey; 4 Faculty of Science, Department of Chemistry, Ankara University, Ankara, Turkey; 5 Gülhane Faculty of Pharmacy, Department of Biochemistry, University of Health Sciences, Ankara, Turkey; 6 Faculty of Science, Department of Chemistry, Hacettepe University, Ankara, Turkey

**Keywords:** Molnupiravir, Molecularly imprinted polymer, Electropolymerization, Photopolymerization, Modified electrode, Differential pulse voltammetry

## Abstract

A comparative analysis of molecularly imprinted polymers based on different synthesis techniques was performed for the recognition of molnupiravir (MOL). The polymerizations were performed with 3-thienyl boronic acid (3-TBA) as a functional monomer by electropolymerization (EP) and with guanine methacrylate (GuaM) as a functional monomer by photopolymerization (PP). Morphological and electrochemical characterizations of the developed sensors were investigated to verify the constructed sensors. Moreover, quantum chemical calculations were used to evaluate changes on the electrode surface at the molecular and electronic levels. The dynamic linear range of both designed sensors under optimized experimental conditions was found to be 7.5 × 10^−12^–2.5 × 10^−10^ M and 7.5 × 10^−13^–2.5 × 10^−11^ M for EP and PP, respectively. The effect of various interfering agents on MOL peak current was assessed for the selectivity of the study. In the presence of 100 times more interfering agents, the RSD and recovery values were determined. The RSD values of GuaM/MOL@MIP/GCE and poly(Py-co-3-PBA)/MOL@MIP/GCE sensors were found to be 1.99% and 1.72%, respectively. Furthermore, the recovery values of the MIP-based sensors were 98.18–102.69% and 98.05–103.72%, respectively. In addition, the relative selectivity coefficient (*k*′) of the proposed sensor was evaluated, and it exhibited good selectivity for MOL with respect to the NIP sensor. The prepared sensor was successfully applied to determine MOL in commercial serum samples and capsule form. In conclusion, the developed sensors provided excellent reproducibility, repeatability, high sensitivity, and selectivity against the MOL molecule.

**Graphical Abstract:**

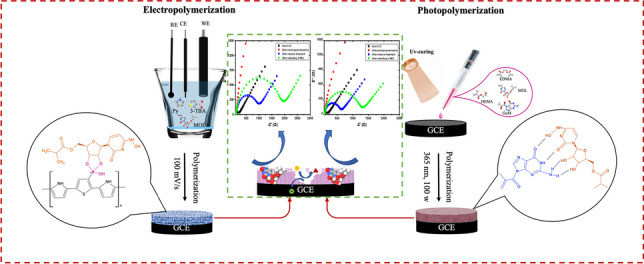

**Supplementary Information:**

The online version contains supplementary material available at 10.1007/s00604-024-06353-w.

## Introduction

The progression to severe symptoms and hospitalization can be significantly reduced with immediate care in confirmed COVID-19 patients with mild symptoms. Molnupiravir (MOL), a potent antiviral drug that may reduce the risk of hospitalization and death rates in non-hospitalized COVID-19 patients, was reported for the effective treatment for SARS-CoV-2 variants of concern (VoCs) [1–3]. The effectiveness statistics in COVID-19 patients have been extensively discussed [4–7]. MOL is an oral antiviral prodrug that has been approved to treat mild-to-moderate COVID-19 who are at high risk of developing severe disease. It has a wide range of preclinical efficacy against RNA viruses, including SARS-CoV-2 and its variations [8–10]. Using sensitive and trustworthy analytical techniques is necessary for the prior to investigating and determining.

In the recent literature, various analytical techniques such as high-performance liquid chromatography (HPLC) and reversed-phase HPLC [11, 12], liquid chromatography-tandem mass spectrometry (LC–MS-MS) [13–15], ultra-performance liquid chromatography-tandem mass spectrometry (UPLC-MS–MS) [16–18], high-performance thin-layer chromatography (HP-TLC) [17], UV–Vis [19], and fluorescent spectroscopy [20] have been reported for the quantification of MOL. The spectroscopy has some disadvantages, such as being only possible for analytes containing chromophores, strongly affected by pH, temperature, pollutants, and impurities, limited dynamic range, and lack of fluorescence of each molecule. However, one of the main drawbacks of chromatography is the high cost associated with the technique, as well as the time-consuming pre-processing steps involved. Electrochemical methods in contrast to chromatographic approaches provide shorter analysis times, minimize the use of organic solvents for green chemistry, and require simple sample preparation. These benefits make electrochemical methods a valuable alternative in various analytical settings, mainly where cost and time efficiency are important considerations. In this context, the determination of MOL using electrochemical methods is available in the more recent literature [21, 22]. However, molecularly imprinted electrochemical sensors have not yet been reported in the literature for the determination of MOL.

MIPs have gained significant attention as a promising technique for achieving excellent selectivity [23, 24]. The sensitivity of MIPs can be significantly improved by carefully considering and optimizing the polymer synthesis, experimental parameters, and surface modifications, which makes them more effective for various analytical and sensing applications [25]. MIPs offer numerous advantages in a wide range of applications with their high selectivity, cheap cost, and excellent chemical and thermal stability. MIPs have played a significant role in various industries and scientific disciplines [26, 27]. Electropolymerization (EP) and photopolymerization (PP) are frequently preferred in the development of functional polymers for drug analysis [23, 28–31]. Using pyrrole as a heteroaromatic monomer in EP demonstrates its suitability due to its biocompatibility and ease of polymerization [32, 33]. These techniques provide researchers powerful tools to create specialized detection systems and structures for drug analysis applications. However, the random orientation of the polymeric chain in this material causes poor adhesion and selectivity, which restrict utility of such kind of sensor systems [34]. Therefore, many different techniques have been proposed to produce copolymers to overcome these limitations and improve the performance of electrochemical sensors [23].

In this study, 3-thienyl boronic acid (3-TBA) and guanine methacrylate (GuaM) monomers were used separately as functional monomers in EP and PP, respectively, to form MIP films with great selectivity and sensitivity for MOL. MIP films were synthesized using GuaM as a functional monomer with 2-hydroxyethyl methacrylate (HEMA), ethylene glycol dimethacrylate (EGDMA), the pore-forming agent polyvinyl pyrrolidine (PVP), and template molecule (MOL) in the PP technique. On the other hand, 3-TBA was polymerized with Py in the presence of MOL in the EP technique. Electrochemical impedance spectroscopy (EIS), cyclic voltammetry (CV), attenuated total reflectance-Fourier transform infrared (ATR-FTIR) spectroscopy, contact angle, and atomic force spectroscopy (AFM) were used to characterize the electrochemical and morphological characteristics of the poly(Py-co-3-TBA)/MOL@MIP/GCE and GuaM/MOL@MIP/GCE sensors. To determine MOL in both capsule form and commercial serum samples, the electrochemical capabilities of the sensors were thoroughly investigated. Furthermore, the template effect and the alterations to the electrode surface were discussed using theoretical calculations.

## Experimental section

### Chemicals and reagents

The detailed information for “chemicals and reagents” was supplied in the supplementary material.

### Instrumentation

The detailed information for “instrumentation” is supplied in the supplementary material.

### Fabrication of the MIP- and NIP-based sensors

The glassy carbon electrode (GCE) was cleaned in methanol/water (1:1, v/v) under sonication for 10 min before each experiment. The alumina slurry was dropped onto the polishing pad before polishing the GCE and then rinsed with ultrapure water and dried at room temperature.

**Scheme 1:**
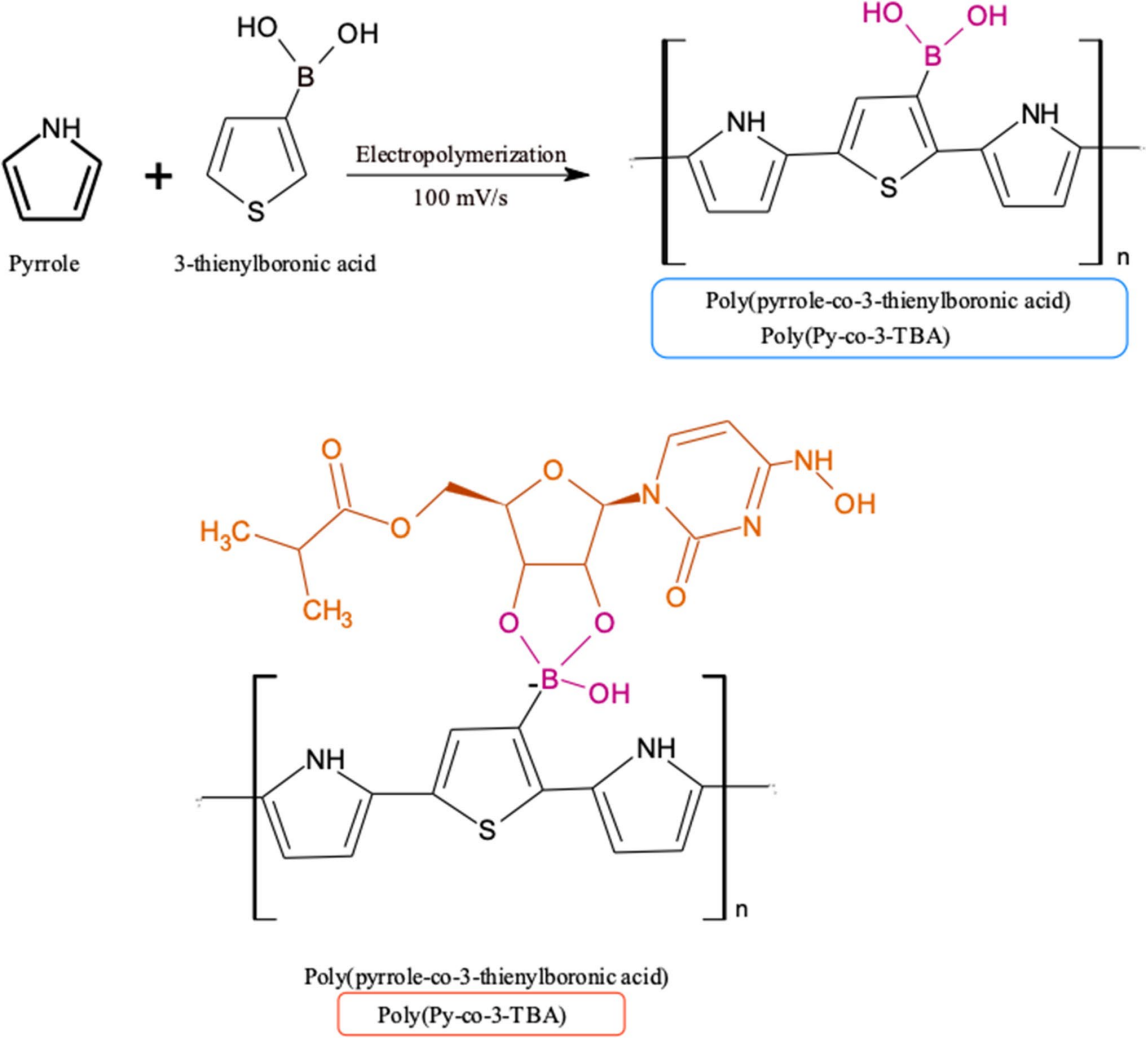
Proposed interaction mechanism between 3-TBA and MOL

**Py** (0.05 M), 3-TBA (0.01 M), and MOL (0.01 M) were dissolved in phosphate-buffered solution (PBS, pH 7.5) containing LiClO_4_ (0.1 M) as supporting electrolyte for the EP process (Scheme 1). The electrode was scanned for 10 cycles of CV from − 0.2 to + 1.6 at a scan rate of 100 mV/s (Figure S1). After polymerization was terminated, the film was washed by ultrapure distilled water. The template molecule was then removed using 7 cycles of CV in PBS (pH 7.5). Then, the electrodes were treated with MOL solution in known concentrations in the ThermoShaker at 500 rpm at 25°C for 10 min to rebind them to the exposed cavities.

**Scheme 2:**
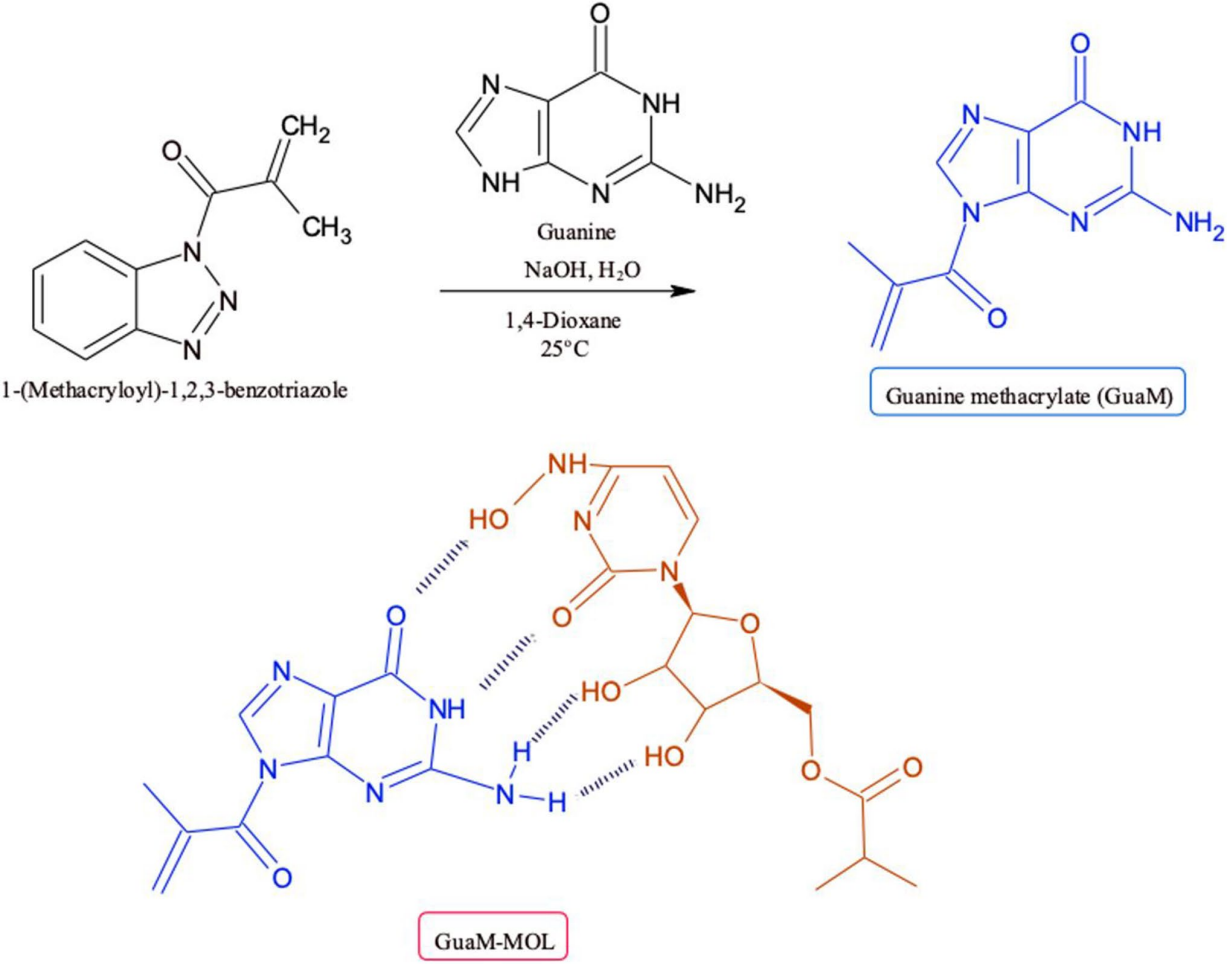
**A** The synthesis route of GuaM and **B** proposed interaction mechanism between GuaM and MOL

From the photopolymerization process, 1 μL of TMSPMA solution was dropped onto the electrode surface and dried in the incubator at 50 °C for 10 min. The silane and methcarylate groups in TMSPM provide bonding between the polymeric film and the electrode surface, preventing the film from being removed from the surface. The PP solution was prepared as follows: homogen solution of pre-polymerization of MOL (0.01 M; template), and GuaM (0.01 M; functional monomer) (detailed information for GuaM synthesis was given in supplementary Materials), 100 μL of HEMA (basic monomer), and 20 μL of EGDMA (cross-linker) was stirred at R.T for 10 min. Twenty microliters of the prepared polymerization solution was taken and 2 μL of 2-hydroxy-2 methylpropisphenone was added (Scheme 2). Subsequently, 0.5 μL of this mixture was dropped directly onto the surface of electrode and polymerization was performed by UV-induced free radical photopolymerization under UV light (100 W, 365 nm) for 5 min at room temperature under nitrogen atmosphere. The MOL template molecule was removed from polymeric coating using a ThermoShaker at 650 rpm at 25°C for 10 min in the presence of the desorbing agent. Nonimprinted polymers (NIPs) were prepared for control studies using the same conditions without adding MOL.

### The commercial serum sample and capsule form applications

The detailed information for “The commercial serum sample and capsule form applications” is supplied in the supplementary material.

### Quantum chemical calculations

The detailed information for “quantum chemical calculations” is supplied in the supplementary material.

## Results and discussion

### Characterization of polymeric films

**Fig. 1:**
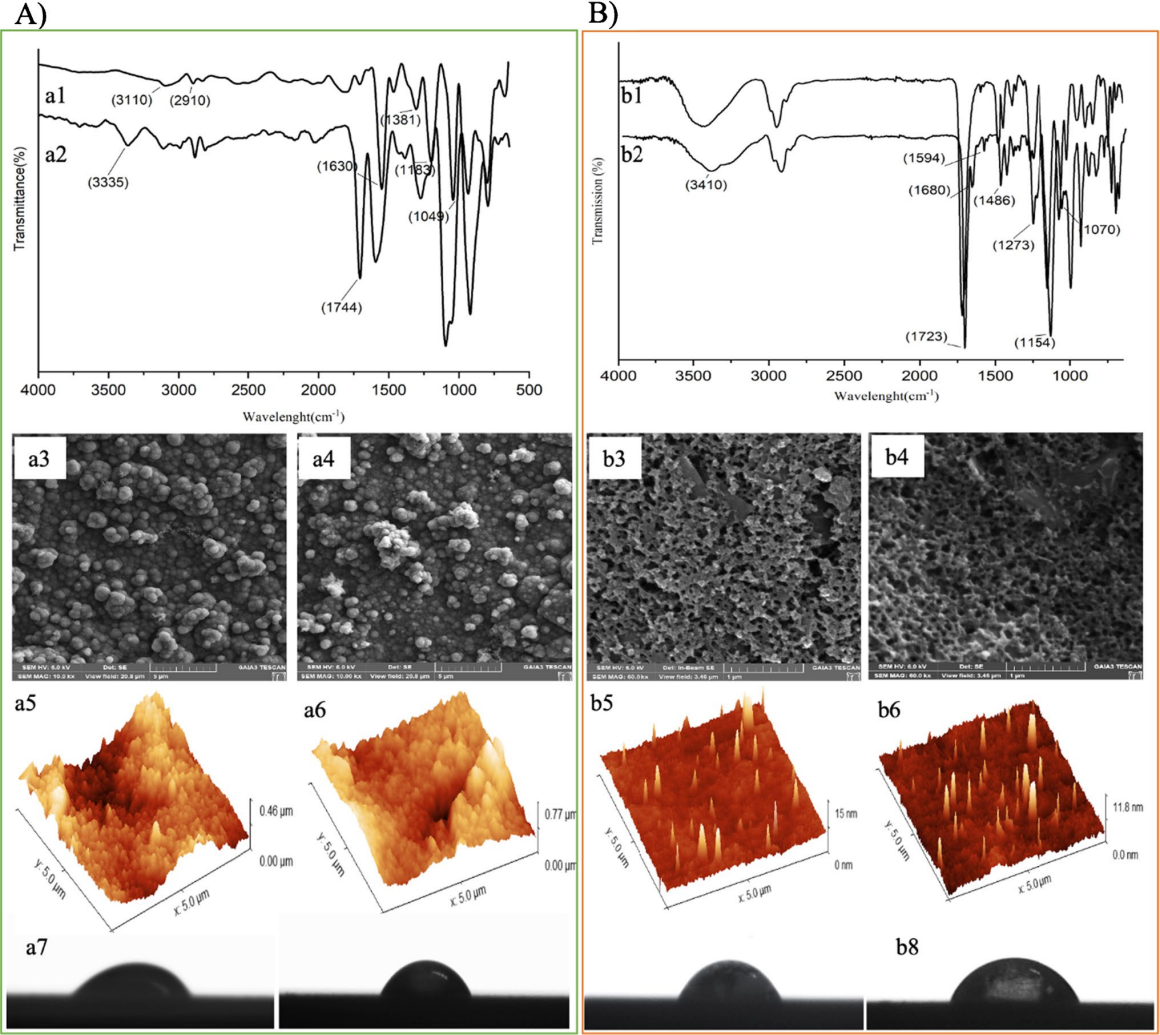
Characterizations of the electrode synthesized by EP **A** and PP **B**. ATR-FTIR spectra of NIP (a1, b1) and MIP (a2, b2); SEM images of MIP (a3, b3) and NIP (a4, b4); AFM images of MIP (a5, b5) and NIP (a6, b6). Contact angle of MIP (a7, b7) and NIP (a8, b8)

The developed sensors’ characterization results are illustrated in Fig. 1. Figure 1(a) displays the FT-IR spectra of fic film for EP. The bands observed at 3110 due to N–H stretching vibration of Py ring and 2910 cm^−1^ are assigned aromatic C–H stretching vibrations of Py and 3-TBA, respectively. The bands at around 1630, 1381, 1183, and 1049 cm^–1^ are typical characteristics of the C = C bonds of thiophene ring, B–O stretching, B–O–H bending, and B–O–H deformation for 3-TBA (Fig. 1(a1)). On the other hand, Fig. 1(a2) presents the strong bands at 3335 cm^−1^ and 1744 cm^−1^ which correspond to O–H and C = O bonds due to inclusion of template molecule in the network of MIP as compared to NIP, which indicates the successful fabrication of NIP- and MIP-based electrochemical sensor films. In Fig. 1(b2), the bands observed for MIP films at 1070, 1154, and 1723 cm^−1^ correspond to the –C–O, –C–O–C, and –C = O vibrations, respectively. Furthermore, a broadband around 3410 cm^−1^ was stemmed from hydroxyl (–OH). The amide I, II, and III bands were seen at around 1594, 1486, and 1273 cm^−1^, which indicates that GuaM was successfully integrated into the polymeric network. The band observed at 1680 cm^−1^ differed, which may be due to the presence of the target molecule in the MIP compared to the NIP (Fig. 1(b2)). When comparing monomers and polymeric chain, for EP and PP, the longer conjugated polymer chain exhibits better symmetry and greater equilibrium electron density than the monomer, and the adsorption peaks of the bond shift to higher/lower wavenumbers (supplementary material S3). As displayed in Fig. 1(a3), the MIP film exhibits a dense and irregular arrangement of particles. Besides, the SEM images of NIP appear to have similar shapes, which is expected as they are synthesized under the same conditions. On the other hand, porous structures were observed for fabricated electrodes synthesized by PP process (Fig. 1(b3, b4)) due to the removal of a sacrificial agent of PVP compared to the electrode synthesized by the EP process. Figure 1(a5, a6, b5, b6) presents typical AFM images of fabricated sensors. The surface deepness for MIP and NIP surfaces were taken as 0.46 μm and 0.77 μm for EP, whereas those were determined as 8.4 nm and 9.8 nm for PP, respectively. These results showed that film formation was successfully achieved and is also well fitted to the results of SEM images. These results also confirm that surface roughness was increased for electrodes synthesized by EP due to the polymerization cycles compared to PP. The contact angle measurement is shown in Fig. 1(a7, a8, b7, b8). The contact angle values obtained on electrode synthesized by EP was 60.5 and 65.0° for MIP and NIP, respectively, while the contact angle value synthesized by PP was 70.5° and 72.8° for MIP and NIP, respectively. The MIP film exhibited a relatively higher hydrophilicity than NIP due to the character of MIPs recognition cavities.

### Electrochemical characterizations of MIP surfaces

The “supplementary materials” file includes “Electrochemical characterizations of MIP surfaces” for this section.

### Optimization of the parameters of the designed sensors

The “supplementary materials” file includes in the detail the “Optimization of the parameters of the designed sensors” for this section.

The parameters of the sensors developed using EP and PP techniques were optimized. Based on the ∆*I* values obtained with both EP and PP, the monomer:template ratio of 1:1 was selected as the optimal value for both sensors in order to generate the most stable and effective polymers. In the EP process, the CV technique was used to create a durable and useful polymeric film. After selecting appropriate monomers and ratios to provide a polymer with the required stability and thickness, the optimal number of EP cycles was found to be 10 cycles. The PP time under UV lamp was optimized, and 0.25 μL of polymerization solution was dropped and exposed to UV light for different periods of time to form a stable polymeric layer on the GCE surface, achieving good reproducible and stable polymerization in 5 min. Appropriate removal solutions were used for both EP and PP. In the EP method, the best peak current value was obtained in PBS solution with pH 7.5 as removal solutions. CV approach was used to remove template molecules after different cycles and the best result was achieved after 7 cycles and this removal solution was used in all steps. In the PP method, 5 M HAc was used as the removal solution. Additionally, different removal times were evaluated and 10 min was chosen as the optimum removal time due to the most stable and reproducible results. In the PP method, 5 M HAc was used as the removal solution. Additionally, different removal times were evaluated and 10 min was chosen as the optimum removal time due to the most stable and reproducible results. To evaluate its effect on rebinding to MIP-based sensors prepared for both EP and PP, the rebinding solution was applied for different times and examined with the help of ThermoShaker (500 rpm, 25°C). When the difference between the peak currents after reconnection and removal was evaluated, the highest peak current values were selected at 10 min for EP and 20 min for PP. The parameters given in Table 1 were optimized to obtain the best poly(Py-co-3-TBA)/MOL@MIP/GCE and GuaM/MOL@MIP/GCE sensors.

**Table 1:** The significant optimization parameters for EP and PP

	poly(Py-co-3-TBA)/MOL@MIP/GCE (EP)	GuaM/MOL@MIP/GCE (PP)
Monomer:template ratio	**1:1**	**1:1**
Dropping volume (μL)		**0.25**
PP time (min)		**5**
Number of cycles for EP	**10**	
Removal solution	**pH 7.5 PBS**	**5 M HAc**
Number of cycles for Removal	**7**	
Removal time (min)		**10**
Rebinding time (min)	**10**	**20**

### Assessment of analytical performance of poly(Py-co-3-TBA)/MOL@MIP/GCE and GuaM/MOL@MIP/GCE sensors

**Fig. 2:**
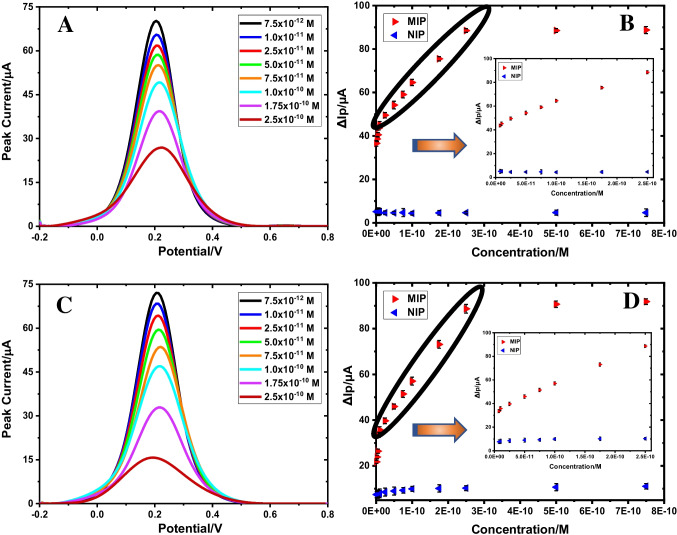
DPV voltammograms obtained after rebinding of various MOL concentrations in standard solution (**A**) and commercial serum solution (**C**), calibration curve for poly(Py-co-3-TBA)/MOL@MIP/GCE in standard solution (**B**), commercial serum solution (**D**) (in 5 mM [Fe(CN)_6_]^3−/4–^solution (0.1 M KCl))

The analytical performance of the poly(Py-co-3-TBA)/MOL@MIP/GCE sensor was assessed by the measurement of MOL in the linear concentration range between 7.5 × 10^−12^ M and 2.5 × 10^−10^ M (Fig. 2B). The regression equation was calculated in this concentration range as Δ*I* (μA) = 1.80 × 10^11^ (μA/M) × C(M) + 44.44 (*r* = 0.996) using 5.0 mM [Fe(CN)_6_]^3−/4−^ solution as the redox probe by indirect measurements. The results of the regression parameters are summarized in Table 1. The LOD and LOQ values were 6.01 × 10^−13^ M and 2.00 × 10^−12^ M, respectively. The LOD and LOQ values were obtained using theoretical calculation methods in the ICH Guidelines. They were calculated based on the “Standard Deviation of the Response and the Slope” method in the ICH Guidelines using the following equations [35, 36]:

$$\mathrm{LOD}\left(\mathrm{LOD}=3\times\frac{\mathrm{standard}\;\mathrm{deviation}}{\mathrm{slope}}\right)\;\mathrm{and}\;\mathrm{LOQ}\left(\mathrm{LOQ}=10\times\frac{\mathrm{standard}\;\mathrm{deviation}}{\mathrm{slope}}\right)$$

The results showed excellent sensitivity from the poly(Py-co-3-TBA)/MOL@MIP/GCE sensor. Under ideal conditions, the proposed sensor was used to identify MOL using the DPV method (Fig. 2A). The performance of the NIP-based sensor was also investigated using poly(Py-co-3-TBA)/MOL@NIP/GCE to determine MOL over the studied concentration range. The MIP curve (red color) showed a linear response with Δ*I* increasing proportional to MOL concentration, but no linearity was observed for NIP. The NIP curve (blue color) exhibited a small Δ*I* value, in contrast to MIP, because there were no NIP-specific recognition sites for MOL. These results indicated the poly(Py-co-3-TBA)/MOL@MIP/GCE sensor has good sensitivity and selectivity for MOL detection.

**Fig. 3:**
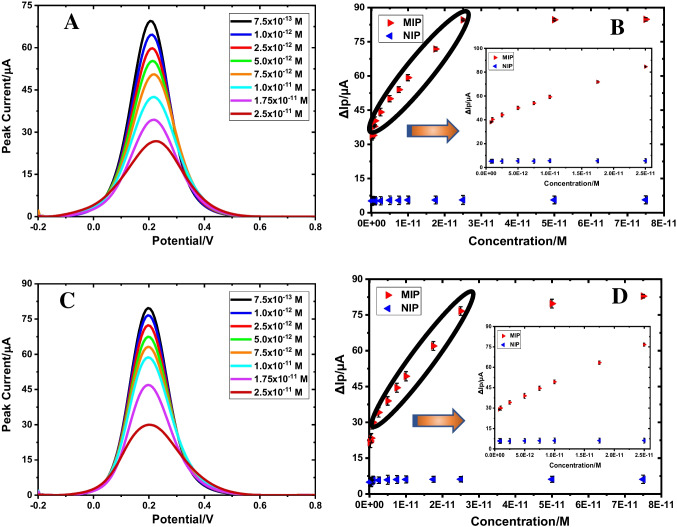
DPV voltammograms obtained after rebinding of various MOL concentrations in standard solution **A** and commercial serum solution **C**, calibration curve for GuaM/MOL@MIP/GCE in standard solution, **B** commercial serum solution **D** (in 5 mM [Fe(CN)_6_]^3−/4–^solution (0.1 M KCl))

The analytical performance of the GuaM/MOL@MIP/GCE sensor was evaluated under ideal experimental conditions with the DPV method using range of MOL concentrations from 7.5 × 10^−13^ M to 2.5 × 10^−11^ M (Table 2). The regression equation was found to be as Δ*I* (μA) = 1.86 × 10^12^ (μA/M) × *C* (M) + 39.11 (*r* = 0.996), and the GuaM/MOL@MIP/GCE sensor displayed a linear response for selective and sensitive MOL detection. The calculated values for the LOD and LOQ were 1.13 × 10^−13^ M and 3.76 × 10^−13^ M, respectively. The NIP-based sensor measured selectivity (blue color) and controlled analytical performance in the same concentration range as the MOL. The relationship between declining [Fe(CN)_6_]^3−/4−^ peaks and rising MOL concentrations is seen in Fig. 3A. The results show good selectivity and sensitivity of GuaM/MOL@MIP/GCE for MOL detection. The plots of MOL concentration versus Δ*I* values for MIP and NIP-based sensors are given in Fig. 3B. The findings demonstrate the superior selectivity and sensitivity of GuaM/MOL@MIP/GCE for detecting MOL. Compared to poly(Py-co-3-TBA)/MOL@MIP/GCE, GuaM/MOL@MIP/GCE showed a porous structure and a thinner layer, enabling the detection of MOL at lower concentrations.

**Table 2:** Regression parameters of MOL on poly(Py-co-3-TBA)/MOL@MIP/GCE and GuaM/MOL@MIP/GCE sensors

	EP		PP	
	Standard solution	Serum sample	Standard solution	Serum sample
Linearity range (M)	7.5 × 10^−12^–2.5 × 10^−10^	7.5 × 10^−12^–2.5 × 10^−10^	7.5 × 10^−13^–2.5 × 10^−11^	7.5 × 10^−13^–2.5 × 10^−11^
Slope (µA/M)	1.80 × 10^11^	2.23 × 10^11^	1.86 × 10^12^	1.95 × 10^12^
SE of slope	6.49 × 10^10^	4.89 × 10^10^	6.74 × 10^10^	4.28 × 10^10^
Intercept (µA)	44.44	33.84	39.11	28.87
SE of intercept	0.77	0.58	0.80	0.51
Correlation coefficient (*r*)	0.996	0.999	0.996	0.998
LOD (M)	6.01 × 10^−13^	1.79 × 10^−12^	1.13 × 10^−13^	6.93 × 10^−14^
LOQ (M)	2.00 × 10^−12^	5.97 × 10^−12^	3.76 × 10^−13^	2.31 × 10^−13^
Repeatability of peak current (RSD%)*	1.07	1.23	0.89	1.11
Reproducibility of peak current (RSD%)*	1.76	1.83	1.62	1.97

^*^Each value is the mean of three experiments

### Application of the developed sensors to commercial serum samples and capsule form

The analysis of MOL has been applied successfully to capsule form and commercial serum samples with the poly(Py-co-3-TBA)/MOL@MIP/GCE and GuaM/MOL@MIP/GCE sensors. The sensor showed excellent performance in complicated matrices with high sensitivity, specificity, and accuracy in the determination (Table 3). The known amounts of the analyte were added to samples during the recovery studies, and the poly(Py-co-3-TBA)/MOL@MIP/GCE and GuaM/MOL@MIP/GCE sensors were used to measure the concentration of the analyte in the samples. The recovery studies were very satisfactory with excellent recovery percentages and RSD values and given in Table 3. The measurements were made with diluted solutions from stock solutions prepared as described in section S1.3. The quantities found without adding known analyte concentrations are given in Table 3 below the label quantity. The findings were found to be consistent with the MOL amount mentioned in the capsule form and commercial serum samples. The MIP-based sensor prepared using the EP method showed a linear response with a proportional increase in Δ*I* with MOL concentration according to DPV data for serum samples. In contrast, the NIP-based sensor showed no significant change in Δ*I* compared to MIP (Fig. 2C, D). The recovery values of the poly(Py-co-3-TBA)/MOL@MIP/GCE sensor for capsule form and commercial serum samples were found as 101.35% and 99.09%, respectively (Table 3). This demonstrated the applicability of the designed sensor for real samples.

**Table 3:** Recovery results for MOL for capsule form and serum samples

	EP		PP	
	Capsule form	Serum sample	Capsule form	Serum sample
Label amount (mg)	200.000	–	200.000	–
Found amount (mg)*	201.778	–	199.013	–
RSD%	1.97	–	1.24	–
Bias%	-0.89	–	0.49	–
Calculated *t*_value_	0.22			
Calculated *F*_value_	0.38			
Spiked amount (mg)	10.000	10.000	10.000	10.000
Found amount (mg)*	10.135	9.909	9.940	10.097
Average recovery (%)	101.35	99.09	99.40	100.97
RSD%	1.50	1.56	1.87	1.60
Bias%	− 0.14	0.91	0.60	− 0.97

*Each value is the mean of five experiments. Theoretical Student *t* and *F* values are 2.13 and 6.38, respectively

Similarly, the MOL was first spiked in capsule form and commercial serum samples at certain concentrations in PP and analyzed with the developed GuaM/MOL@MIP/GCE sensor and results are given in Fig. 3C and D. The recovery percentages were calculated as 99.40% and 100.97%, respectively (Table 3). These findings also demonstrate that the GuaM/MOL@MIP/GCE sensor has been successfully used in real samples.

The Student *t*- and *F*-tests were used to statistically evaluate the validity of the results obtained from the poly(Py-co-3-TBA)/MOL@MIP/GCE and GuaM/MOL@MIP/GCE sensors. The experimentally obtained Student *t*- and *F*-test values at the 95% confidence level are lower than the theoretically stated Student *t*- and *F*-tests, demonstrating the relevance of these two recommended sensors in MOL studies.

### Selectivity studies

The ability to achieve greater selectivity and affinity for the target molecule is the main benefit of integrating MIPs into electrochemical sensors. To emphasize this benefit and demonstrate the sensor’s selectivity in comparison to other compounds with comparable structures (such as ribavirin (RIB), lamivudine (LAM), zidovudine (ZID), zalcitabine (ZAL), and emtricitabine (EMT)), *relative selectivity coefficient* (*k′*) tests were conducted. The ratio of the Δ*I* values of the template molecule (MOL) to those of the competing drugs (RIB, LAM, ZID, ZAL, and EMT) was used to calculate the selectivity coefficient (*k*) value. The selectivity coefficients of MOL for MIP/GCE and NIP/GCE were also calculated to estimate the relative selectivity coefficient (*k*′), which indicates the specific recognition capability gained by MIPs (Table 4). “The selectivity coefficient (*k*) is defined as the ratio between current value for template and structurally analog drugs as follows: *k* = current value _template_/current value_competitor_ shows that selectivity (*k*) found for MOL was greater; this implies that MIP has relatively better binding tendencies with template MOL as compared to other compounds. The relative selectivity coefficient (*k*′) can be defined as expressed in Eq. *k*′ = *k*_MIP_/*k*_NIP_ (Table 4). This equation allows an estimation of the effect of imprinting on selectivity which measure the identification capability and selectivity of the MIPs for template with respect to NIPs [23, 29, 37]. A high *k*′ value confirmed that the imprinting process was achieved successfully and provided specific recognition sites toward MOL based on the size and shape of the template’s molecules. According to these values, poly(Py-co-3-TBA)/MOL@MIP/GCE specifically recognized MOL molecules by 3.94-, 5.43-, 5.62-, 6.10-, and 6.12-fold corresponding to RIB, LAM, ZID, ZAL, and EMT, respectively. For the GuaM/MOL@MIP/GCE, these values were calculated as 4.02-, 6.93-, 6.86-, 7.35-, and 7.49-fold for RIB, LAM, ZID, ZAL, and EMT, respectively Also, the *k*′ values were greater than 1.0, which is the threshold value for indicating resolution between the target and competitor molecules.” As a result, both poly(Py-co-3-TBA)/MOL@MIP/GCE and GuaM/MOL@MIP/GCE surfaces exhibited higher selectivity toward MOL than RIB, LAM, ZID, ZAL, and EMT compared to NIP surfaces. GuaM/MOL@MIP/GCE also displayed greater selectivity for MOL than poly(Py-co-3-TBA)/MOL@MIP/GCE.

**Table 4:**
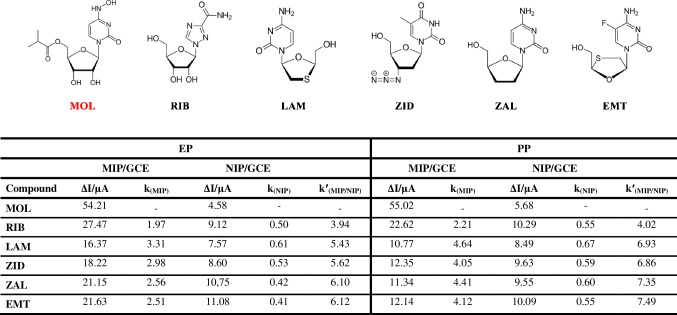
Selectivity values of MOL and other similar drug substances

### Interference studies

**Fig. 4:**
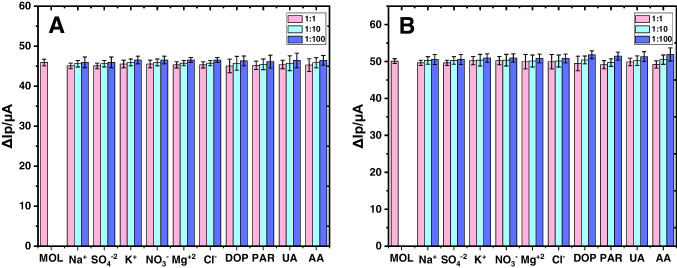
Bar graphs of **A** 5.0 × 10^−11^ M MOL at poly(Py-co-3-TBA)/MOL@MIP/GCE; **B** 5.0 × 10^−12^ M MOL at GuaM/MOL@MIP/GCE in the presence of interfering agents at 1:1, 1:10, and 1:100 ratio using 5 mM [Fe(CN)_6_]^3−/4−^ redox probe

The performance of MIP-based sensors can be affected by various substances present in biological fluids, such as K^+^, Cl^–^, Na^+^, SO_4_^2–^, dopamine (DA), ascorbic acid (AA), uric acid (UA), and paracetamol (PAR) in the serum matrix. The MOL concentrations employed for the selectivity test were 5.0 × 10^−11^ M and 5.0 × 10^−12^ M, respectively, in the poly(Py-co-3-TBA)/MOL@MIP/GCE and GuaM/MOL@MIP/GCE sensors. The RSD and recovery values were calculated in the presence of 100 times more interfering agents. Hereby, RSD values for poly(Py-co-3-PBA)/MOL@MIP/GCE and GuaM/MOL@MIP/GCE sensors were determined as 1.72% and 1.99%, respectively. Additionally, the MIP-based sensors’ recovery values were found to be 98.18–102.69% and 98.05–103.72%, respectively (Fig. 4). These results demonstrated that interfering substances showed low or no effect on the effectiveness of sensors designed for MOL detection.

### Quantum chemical calculations

**Fig. 5:**
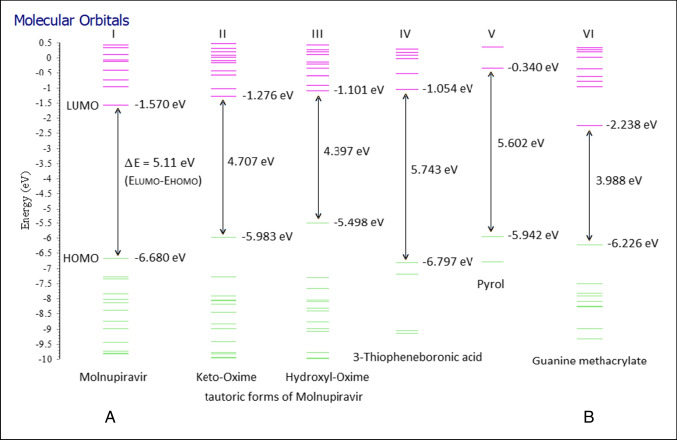
Comparison of HOMO–LUMO molecular orbital energy diagrams.** A** Δ*E*_(LUMO–HOMO)_ energy of trio MOL(I, II, and III), 3-TBA (IV) and Py (V) = 3.928 eV [23]. **B** ΔE_(LUMO–HOMO)_ energy of duo MOL(I, II, and III) and GuaM (VI) = 3.260 eV

**Fig. 6:**
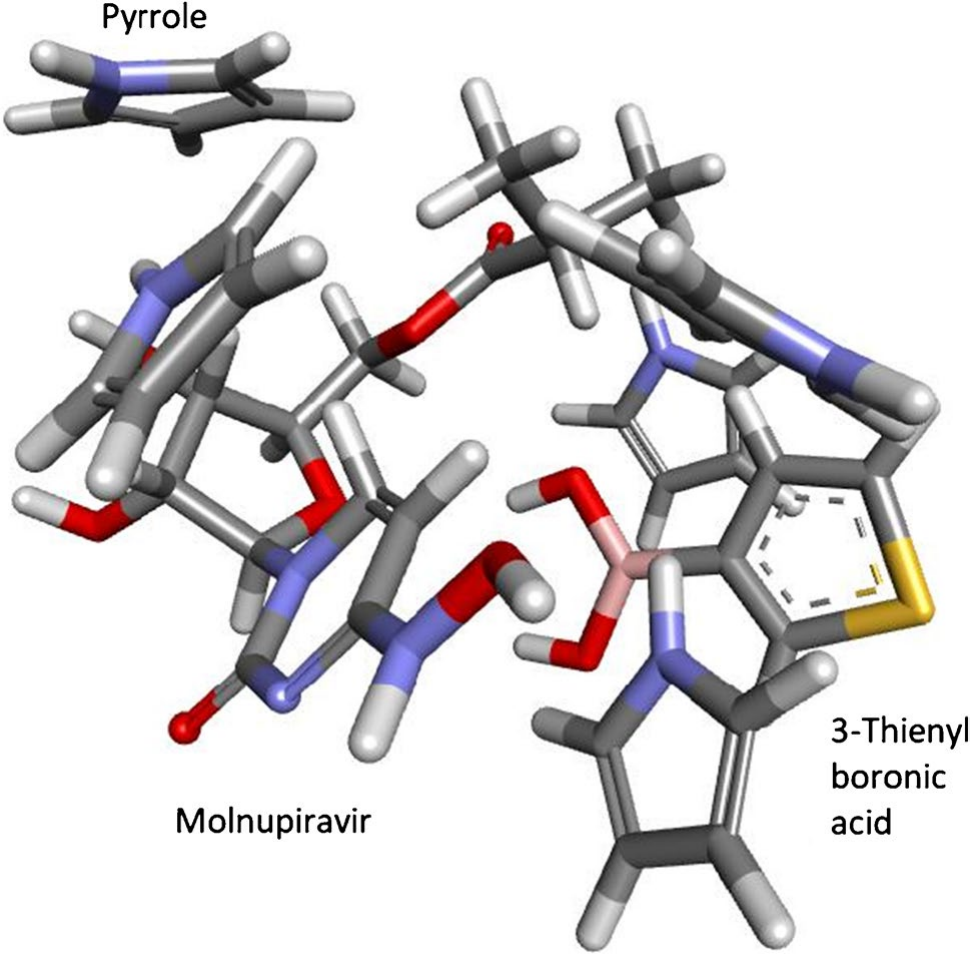
Schematic representation of the molecular docking results of five pyrroles around the poly(Py-co-3-TBA)/MOL complex

The structures of the MOL, 3-TBA, and GuaM molecules used in this study (A), the calculated optimized structure (B), and the electrostatic potential (ESP) charge distribution on the surface of the molecule (C) were subjected to quantum chemical calculations in order to evaluate the applicability of the sensors (Figures S5-S6-S7). Additionally, computer computational analysis of MOL has two stable conformations: keto oxime and hydroxyl oxime [38]. The calculations for these molecules are given in Figure S5-III and Figure S5-IV, respectively. Molecules are polar, as seen on ESP maps (red, electronegative; blue, electropositive). As a result, they can serve as electron donors and acceptors, or they can create non-covalent or hydrogen bonds. Additionally, it appears that MOL’s stable conformations are polar (Figure S5-III and Figure S5-IV). On the other hand, it can be concluded from a comparison of the energy levels of the compounds (Fig. 5) that the addition of 3-TBA and Py [23] to MOL (1:5:1) raises the energy level of the high-occupied molecular orbital (HOMO) (from − 6.680 to − 5.942 eV). The GuaM contribution (1:1) also increased the HOMO energy level from − 6.680 to − 6.226 eV, whereas it decreased the LUMO energy level from − 1.570 to − 2.238 eV. This means that the monomers used show HOMO and LUMO dope properties against MOL. On the other hand, when the stable conformations of MOL are taken into consideration, it is seen that these properties do not change. However, the novel polymeric forms (poly(Py-co-3-TBA)/MOL@MIP/GCE and GuaM/MOL@MIP/GCE) have higher electron mobilities due to the smaller band gap, which makes the template effect determination easier. In Table S1, molecules’ computed electronic, energetic, and geometric descriptor values are presented. According to the first view, MOL molecules have bigger geometric descriptor values for SASA (solvent accessible surface area) and SA (surface area) than other molecules, which will cause their solubility in the polymeric matrix to occur more quickly. Additionally, the findings of the calculations for a molecule’s solubility (LogS) (Table S1) and electronic descriptors (absolute electronegativity, *χ*; extra electronic charge from the environment, and ΔNmax) were consistent. On the other hand, electronegativity causes areas of positive and negative charge to form on the surface of the molecule, and this induced charge has an impact on the molecule’s solubility in solvents. Thus, as solvent-molecule polar-polar interactions grow, a polar molecule dissolves readily in a polar solvent. MOL is more soluble because it has stronger polar SASA and SA values than other molecules. Additionally, MOL’s hydrophobicity descriptor LogP is smaller than that of other molecules, suggesting that MOL is more hydrophilic and dissolves more quickly as a result. On the other hand, the poly(Py-co-3-TBA)/MOL@MIP pair’s docking calculation with Py reveals that up to 5 Py molecules can be attached to the structure (Fig. 6 and Figure S8), supporting the findings of the experiment (Figure S3A). The ideal GuaM/MOL@MIP form ratio was likewise determined to be 1:1 (Figure S9), which was also supported by an experiment (Figure S4A) (MOL-GuaM; 1:2, *E* =  − 74668.127477 eV; 1:1, *E* =  − 53644.244489 eV). This finding demonstrates that a 1:2 increase in stability results in an unacceptable reduction in the MOL’s resolution (Figure S9).

### Comparison with other methods

The “Comparison with other methods” is included in the “supplementary materials” file for this section.

## Conclusion

In this study, different types of MIP-based sensors were constructed for detection of MOL in standard solution, capsule form, and commercial serum samples. Py and 3-TBA monomers were co-polymerized to create the poly(Py-co-3-TBA)/MOL@MIP/GCE sensor for EP, while GuaM and HEMA were co-polymerized to create the GuaM/MOL@MIP/GCE sensor for PP. The poly(Py-co-3-TBA)/MOL@MIP/GCE senor showed shortest response time that of the GuaM/MOL@MIP/GCE sensor when optimization parameters were taken into account. However, GuaM/MOL@MIP/GCE sensor showed better performance for the LOD, LOQ, repeatability, reproducibility, and reusability than the poly(Py-co-3-TBA)/MOL@MIP/GCE sensor. Furthermore, commercial serum samples and capsule forms were used to test the applicability of the created sensors. The accuracy and usefulness of the sensors were demonstrated by the recovery and %RSD values. The selectivity was calculated using the *k*′ values of the substances with similar drugs. Additionally, sensor’s selectivity was not impacted by the presence of interfering agents at a concentration 100 times that of the target molecule. As a result of the stability studies, the sensor developed by EP method was stable for 2 days, while the sensor prepared by PP method was stable for 5 days. In light of these data, it can be concluded that the sensor created for MOL using PP has a better stability.

The accuracy of the sensors created using quantum chemical calculations was thoroughly discussed, and electrochemical and morphological characterizations confirmed MIP-based sensors. As a result, the GuaM/MOL@MIP/GCE sensor compared to poly(Py-co-3-TBA)/MOL@MIP/GCE offered significant advantages such as linear range, LOD and LOQ values, stability, reusability, excellent selectivity, and easy applicability. The accuracy of sensors created using quantum chemical calculations was investigated.

### Supplementary Information

Below is the link to the electronic supplementary material.

Supplementary file1 (DOCX 2647 KB)

## Data Availability

The datasets generated during and/or analyzed during the current study are available from the corresponding author on reasonable request.
