# Supplementary material for: A comparative study of electropolymerization and photopolymerization for the determination of molnupiravir and their application in an electrochemical sensor via computationally designed molecularly imprinted polymers
Source: Mikrochim Acta. 2024 Apr 17;191(5):270. doi: 10.1007/s00604-024-06353-w (PMC11024036; doi:10.1007/s00604-024-06353-w)
Supplement: Supplementary file 1 — Supplementary file1 (DOCX 2647 KB) [file 604_2024_6353_MOESM1_ESM.docx]

**Electronic Supplementary Material**

**A comparative study of electropolymerization and photopolymerization for the determination of molnupiravir and their application in an electrochemical sensor via a computationally designed molecular imprinted polymers**

Ahmet Cetinkaya^1,2^, M. Altay Unal^3^, Hasan Nazır^4^, M. Emin Çorman^5^, Lokman Uzun^6^,

Sibel A. Ozkan^1^*

*^1^Ankara University, Faculty of Pharmacy, Department of Analytical Chemistry, Ankara, Türkiye*

*^2^Ankara University, Graduate School of Health Sciences, Ankara, Türkiye*

*^3^Stem Cell Institute, Ankara University, Balgat, Ankara, Türkiye*

*^4^Ankara University, Faculty of Science, Department of Chemistry, Ankara, Türkiye*

*^5^University of Health Sciences, Gülhane Faculty of Pharmacy, Department of Biochemistry, Ankara, Türkiye*

*^6^Hacettepe University, Faculty of Science, Department of Chemistry, Ankara, Türkiye*

### **S1. Experimental section**

### **S1.1. Chemicals and reagents**

Molnupiravir (MOL) and Viron capsules were supplied from Optimus Drugs in India. The active component for ribavirin was obtained by Sanovel Pharm. Company in Istanbul, Turkey. Which used for the polymerization process in both electropolymerization (EP) and photopolymerization (PP) techniques, pyrrole, 3-thiophenyl boronic acid (3-TBA;97%), 2-hydroxyethyl methacrylate (HEMA; ≥99%), ethylene glycol dimethacrylate (EGDMA; >98.0%) and 2-hydroxy-2-methylpropiophenone (>97%), were obtained from Sigma-Aldrich. Phosphoric acid (>85.0%), disodium hydrogen phosphate (Na_2_HPO_4_), sodium dihydrogen phosphate (NaH_2_PO_4_), lithium perchlorate (LiClO_4_), sodium acetate trihydrate (>99%), acetic acid (≥99%), sodium hydroxide (NaOH), methanol, boric acid (≥99%) and 1-(methacyroyl)-1,2,3-benzatriazole (MA-Bt) were used in the preparation of support electrolyte solutions, these were purchased from Sigma-Aldrich. Moreover, zidovudine, zalcitabine, lamivudine (≥98), emtricitabine (≥98), paracetamol, ascorbic acid, potassium nitrate (KNO_3_; ≥99.0%), sodium sulfate (Na_2_SO_4_;≥99.0%), magnesium chloride (MgCl_2_; ≥98.0%), dopamine hydrochloride, and drug-free commercial serum samples were used for studies showing the selectivity and applicability of the sensors, and this active compounds were provided by Sigma Aldrich. Both potassium ferricyanide (K_4_[Fe(CN)_6_],≥99.0%) and potassium ferrocyanide (K_3_[Fe(CN)_6_], ≥98.5%) were purchased from Merck. No pretreatment was applied to the extremely pure materials utilized in the experiments. The refrigerator was set to 4°C and used to keep all prepared solutions.

### **S1.2. Instrumentation**

Cyclic voltammetry (CV), differential pulse voltammetry (DPV), and electrochemical impedance spectroscopy (EIS) measurements were made using AUTOLAB (NOVA 2.1.5 software). Electrochemical measurements were performed in a three-electrode cell using a saturated Ag/AgCl (3 M KCl) electrode as the reference electrode, a Pt wire as the counter electrode, and GCE as the working electrode. A glass electrode combination pH meter (Mettler-Toledo pH/ion S220, Switzerland) was used for pH measurements of the prepared solutions, and a precision balance (Ohaus Instruments, Shanghai, China) was used for weighing the all ingredients. Photopolymerization (PP) was carried out using a UV light (100 W, 365 nm). A thermo-shaker (Biosan TS-100) was used for both removal and rebinding of the target molecule in the developed sensors, and all experimental studies were carried out at room temperature (25 ^o^C).

FTIR analyzes were performed using the Shimadzu 8000 series (Shimadzu, Japan) using attenuated total reflection mode (ATR-FTIR). The scanning was performed in the mid-infrared region (4000 to 650 cm^–1^). The surface morphology of the electrodes was examined by scanning electron microscopy (SEM, TESCAN GAIA 3, Brno-Kohoutovice, Czech Republic), and the surface deepness were taken by atomic force microscopy (AFM) in semi-contact mode in air atmosphere (Nanomagnetics Instruments (Oxford, England). Contact Angle measurements were also obtained with Krüss DSA100 (Hamburg, Germany).

**1^st^**

**10^th^**

**Figure S1.** CV of the EP process in the mixture of 0.01 M MOL, 0.05 M Py, and 0.01 M 3-TBA solution with 10 cycles at a scan rate of 50 mV/s.

**Synthesis of guanin methacrylate (GuaM) functional monomer**

Guanine methacrylate (GuaM) monomer was yielded to be used as a functional monomer in the photopolymerization technique according to previous literature [1]. Briefly, guanine (0.01 mol) was dissolved in aqueous solution of NaOH (1.0 M). 1-(methacyroyl)-1,2,3-benzatriazole (MA-Bt, 5.52 mmol) was dissolved in 4-dioxane (25 mL) solution and added slowly to guanine solution under ambient atmosphere. After the reaction was completed, the residue was diluted with water and 1H-benzotriazole was removed with ethyl acetate (3× 50 mL). Finally, the GuaM monomer was yielded after removing water (5.0 mL) with a rotary evaporator.

### **S1.3. The commercial serum sample and capsule form applications**

By measuring the MOL in both capsule form and commercial serum samples, the accuracy of the poly(Py-co-3tBA)/MOL@MIP/GCE and GuaM/MOL@MIP/GCE sensors were examined. Five tablets containing 200 mg MOL were weighed for recovery studies. To prepare the 10 mM MOL capsule stock solution, the calculated amount was weighed using a precision balance and the resulting amount was dissolved in methanol. The prepared solution was kept in an ultrasonic bath for 15 minutes and then centrifuged at 5000 rpm for 20 min. A series of intermediate stock solutions were prepared using the main stock solution. Afterwards, rebinding solution of known concentration was prepared using intermediate stock solution and diluted with ultrapure water. As a result, standard calibration data was used to determine the MOL in capsule form.

The prior to the investigations, serum samples that had been stored in a -20°C freezer were dissolved at room temperature (25°C). 1.0 mL of 10 mM MOL, 3.6 mL of serum sample, and 5.4 mL of acetonitrile were taken into a test tube and diluted. The produced serum solution was first sonicated for 15 min. Then, to precipitate protein residues, the resulting serum solution was centrifuged at 5000 rpm for 20 min. Serum calibration plots were obtained using the linearity ranges found for both sensors. Recovery studies were carried out by spiking the pure MOL solution to certain concentrations and relative standard deviation (RSD) values were calculated for each concentration.

### **S1.4. Quantum chemical calculations**

In theory, it is possible to anticipate the physicochemical behavior of molecules by computing their basic features, such as their energy, bonding, and polarization. The Gaussian 2016 (Revision C.01) program package was used in this investigation to determine the fundamental characteristics of molecules utilizing the hybrid Density Functional Theory (DFT/B3LYP) approach [2]. Molecule optimization and natural bond orbital (NBO) analyses are part of the calculations. For NBO analysis, a basis set called 6-311++G(d,p) was employed, whereas optimization calculations were performed using a basis set called 6-31G(d). After each molecule’s optimization calculation, frequency analysis calculations were also carried out at the same theoretical level, and the fictional frequency number, NImag-local minimum (NImag=0), was used to confirm the stabilization of the optimized structures. The computation results were calculated using the GaussView (ver. 6.1.1) and Chemissian (ver. 4.38) programs, both of which are a component of the Gaussian program suite [3]. Moreover, to further investigate the interaction of MOL with polar solvent molecules, in other words its template property, certain electrical (chemical potential, additional electronic charge from the environment), energetic (electron affinity, ionization potential, electronegativity), geometric (surface area, solvent accessible surface area) and solubility descriptors were computed (using the programs Gaussian 2016 and BIOVIA Discovery Studio 2022) [2,4]. (**Table S1**). In this calculation, a short internal unifying program was utilized together with the two Py modules prepare_ligand4.py and _receptor4.py from the AutoDock (version 4.2.6) software package and the AutoDock-Vina (version 1.2.5) program [5]. The box dimensions in the blind docking calculation were set to about three times the expected diameter of the ternary molecule (30x29x24 Å) (**Figure S5**) to achieve the maximum number of Py molecules (coordinates and box dimensions respectively (Å): center; x= -0.30, y= -0.181, z= 0.789; size; x= 30, y= 29, z= 24). The docking calculation results were shown and assessed by the BIOVIA Discovery Studio 2022 [5] application (**Figure S5-B**). In this investigation, the three-dimensional structures of MOL, **Py, and 3-TBA** were retrieved from the PubChem database (PubChem CID: 145996610, 14991078, and 581760, respectively) and used as input data in the computations. Additionally, **Py**'s quantum chemical and QSAR values are taken from the reference [6].

### **S2. Results and Discussion**

### **S2.1. Electrochemical characterizations of MIP surfaces**

Electrochemical characterizations should be used to examine the conductivity and charge transfer properties of the prepared MIP-based GCE surfaces. Here, CV and EIS are the most effective and important techniques to fully reveal the electron transfer processes on unmodified and modified electrode surfaces. Consequently, both were used to perform measurements in 5.0 mM [Fe(CN)_6_]^3-/4-^ solution, which served as a redox probe for electrochemical characterization.

Firstly, CV measurements were performed (after polymerization, after removing MOL, and after reattaching) on bare GCE, poly(Py-co-3-TBA)/MOL@MIP/GCE and GuaM/MOL@MIP/GCE sensors (**Figure S2A** and **Figure S2B**). It was found that the largest peak current values (***black line***) of the redox probe correspond to the bare GCEs due to the absence of a polymeric coating on the surface. On the other hand, the formation of an insulating polymeric coating on the GCE surface, which prevented active and efficient electron migration, prevented the peak current values (***red line***) of the redox probe from appearing after polymerization. Following the removal of MOL, the structure of the MIP film forms particular binding gaps that enable the acceleration of electron transfer and an increase of the peak current values (***blue line***) of the redox probe. The peak current values (***green line***) of the redox probe decline as expected as a result of MOL's rebinding.

The same procedures were used to analyze Nyquist plots and changes in charge transfer resistance (Rct) in EIS measurements (**Figure S2B**, **Figure S2D**, and **Table S2**). EIS data shows that the bare GCE surface (**black dots**) has the lowest Rct values due to easy electron conduction. After polymerization, Rct values (**red dots**) reach the largest value because polymeric coatings that prevent electron transport are formed on the GCE surface. When MOL molecules are removed, special gaps are formed on the MIP surfaces and Rct values (**blue dots**) decrease when electron transfer occurs. However, after rebinding’s of MOL, the holes are partially filled, electron transport becomes difficult, and Rct values (**green dots**) increase again. This suggests that MOL molecules bind only to specific cavities in the designed MIP sensors.

**D**

**B**

**A**

**C**

**Figure S2.** (A) CV, and (B) EIS measurements of bare GCE, after EP, after removal treatment, and after rebinding of MOL for poly(Py-co-3-TBA)/MOL@MIP/GCE; (C) CV, and (D) EIS measurements of bare GCE, after PP, after removal treatment, and after rebinding of MOL for GuaM/MOL@MIP/GCE in 5 mM [Fe(CN)_6_]^3−/4–^ redox probe.

### **S2.2. Optimization of the parameters of the designed sensors**

***Monomer: template ratio***

To create stable and effective polymeric films, the monomer: template ratio must be optimized. The ideal ratio should be established because interactions between the monomer and the template directly affect how polymeric films develop. The monomer: template ratio also increases the selectivity of sensors in molecular imprinting techniques. However, excessive use of the monomer may lead to a non-selective electrochemical reaction for the template, which may also cause the imprinting sites to be distorted. The various monomer: template ratios (1:1, 2:1, 3:1, 4:1, and 5:1) were employed in this situation for polymeric films made with EP and PP, and the differences between the peak currents obtained after removal and after polymerization were calculated (**Figure S3A** and **Figure S4A**). In order to obtain the most effective and stable polymers, the monomer: template ratio was chosen as 1:1, the best value for both sensors, based on the ∆I values obtained with both EP and PP. The potential interactions of well-matched functional groups between MOL and functional monomers (3-TBA or GuaM) were selected considering their structural properties to form a complementary structure similar to the key-lock relationship in order to achieve the highest level of specificity and selectivity. These findings clearly supported the study's selection criteria.

***Number of cycles for EP***

The EP process is essential for producing a robust and practical polymeric film. After selecting the proper monomers and ratios, the CV technique was fabricated with 5, 10, 15, 20, and 25 cycles to obtain a polymer with the desired thickness and stability. The effectiveness of the assay was evaluated by taking the differences between the peak current values of after removal and after EP. The difference between peak currents was achieved as a maximum value at 10 cycles and remained unchanged after 20 cycles. Therefore, the most reliable and effective number of cycles for EP was determined to be 10 cycles (**Figure S3B**).

***Dropping volume***

The stability and thickness of the MIP film are also affected by the amount of monomer solution dropped onto the GCE surface. In addition, as will be mentioned below, it is one of the important criteria in terms of the duration of the polymerization process. Here, the amount of dripping volume was evaluated using different volumes between 0.25 and 1.50 µL, and the peak current values between after removal and after PP were calculated. According to the peak current values obtained, the best dripping volume amount was found to be 0.25 µL (**Figure S4B**).

***PP Time***

A UV lamp with wavelength and power density of 365 nm and 100 W, respectively was used to construct the Poly(Py-co-3-TBA)/MOL@MIP/GCE sensor. A stable polymeric layer was formed on the GCE surface by optimizing the PP time under the UV lamp. The GCE surface was exposed to UV light for 5, 7, 10, 15, and 20 min following application of 0.25 μL of polymerization solution. The best PP time was determined by calculating the differences between the peak current values of after removal and after PP. It was found that good reproducible and stable polymerization could be achieved within 5 min (**Figure S4C**).

***Removal solutions and removal step***

A critical stage in the process is to remove the template molecules from MIP films without harming the polymeric film. Following the removal of the template molecule in this step, a particular cavity is created, and the analyte is easily bonded to this cavity. For both EP and PP, appropriate removal solutions were used. In the EP method, PBS (for pH 6.0, 7.0, 7.5, and 8.0) and Britton-Robinson buffer (BRB, for pH 9.0) were used as removal solutions. The best peak current value was obtained in PBS solution with pH 7.5. This buffer was mixed with sodium chloride (NaCl, 0.1 M), methanol (MeOH), acetonitrile (ACN) and acetone in a 1:1 volume ratio and tested to see how well it could remove MOL. The CV approach was used to eliminate the template molecules after 1, 3, 5, 7, and 10 cycles. As can be observed in **Figure S3D**, the best outcome was attained after 7 cycles, and this removal solution was used in all steps. The effects of various acidic/basic solutions and organic solvents (1M HCl, 15M HAc, 5M NaOH, methanol, acetonitrile and acetone) were evaluated in the PP method. The highest ΔI value was obtained with 5M HAc, and the extraction time was optimized with this solution (**Figure S4D**). Here, removal times of 5, 10, 15, 20 and 25 min were evaluated and 10 min was decided as the optimum removal time due to the most stable and reproducible results observed (**Figure S4E**).

***Rebinding process***

The rebinding process is an important parameter determining the analysis time and performance. Therefore, the MIP-based sensor prepared for EP was immersed in a 5x10^-10^ M MOL solution to evaluate its effect on reattachment at different times (3, 5, 7, 10, and 15 min) and was examined using ThermoShaker (500 rpm, 25^o^C). When the difference between the after-rebinding and after-removal peak currents was evaluated, it was seen that ΔI remained almost the same after reaching the highest value at 10 min. Therefore, according to **Figure S3E**, the rebinding time was selected as 10 min for the poly(Py-co-3tBA)/MOL@MIP/GCE sensor.

The same steps followed for EP were performed for PP. The MIP-based sensor prepared for PP was immersed in 5.0x10^-12^ M MOL solution to evaluate its effect on rebinding at different times (5, 10, 15, 20, and 25 min) and performed using ThermoShaker (500 rpm, 25^o^C). By calculating the difference between the peak currents after rebinding and after removal, the optimal rebinding time was found for stable and efficient binding. According to **Figure S4F**, the rebinding time for the GuaM/MOL@MIP/GCE sensor was selected as 20 min.

**BB**

**A**

**CB**

**EB**

**DB**

**Figure S3.** The plots of ΔIp values of DPV (A) template: monomer ratio, (B) EP number of scan, (C) removal solutions, (D) removal number of scan, (E) rebinding time for EP.

**BB**

**AB**

**DB**

**CB**

**F**

**EB**

**Figure S4.** The plots of ΔIp values of DPV (A) template: monomer ratio, (B) dropping volume, (C) polymerization time, (D) removal solutions, (E) removal time, and (F) rebinding time for PP.

| **Molnupiravir (MOL, template molecule)** |
| --- |
| 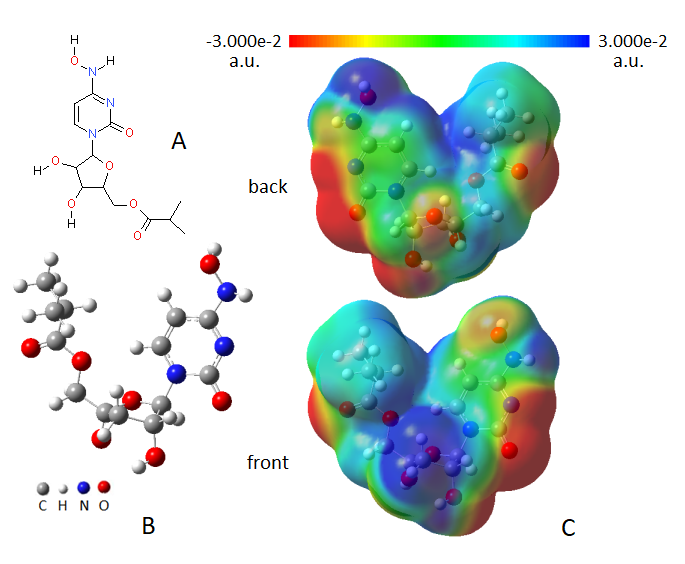  I |
| 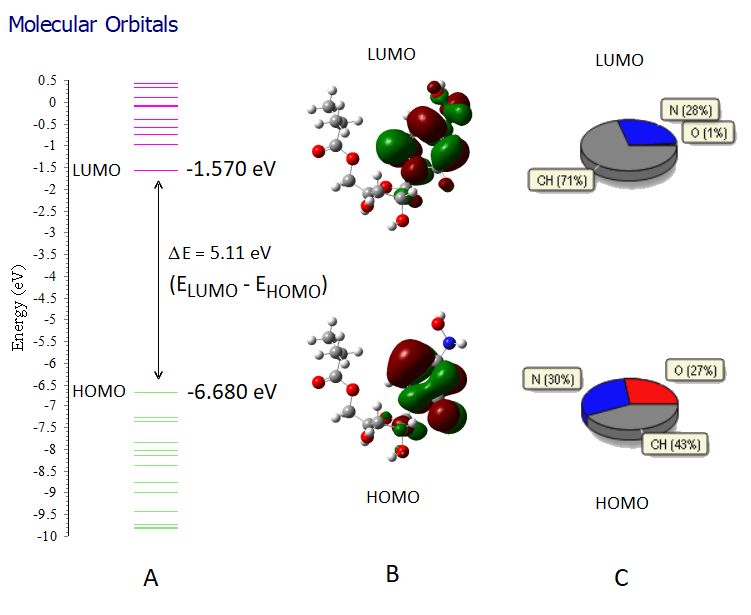  II |

| **Keto-Oxime tautomeric form** |
| --- |
| 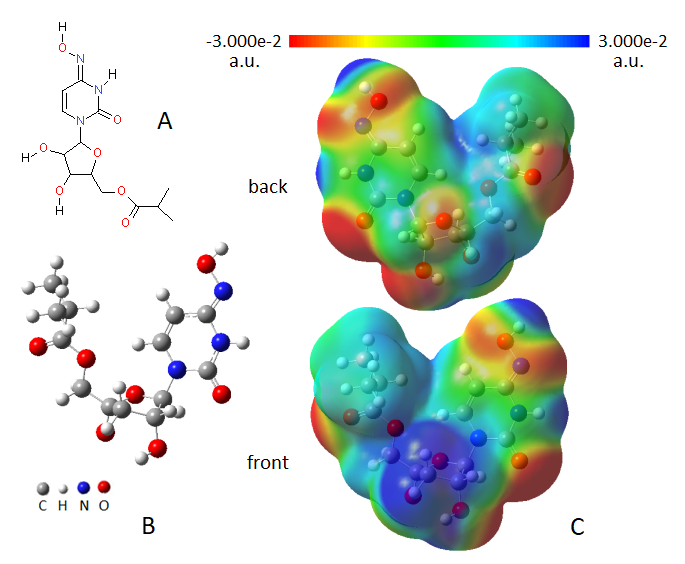  III |
| **Hydroxyl-Oxime tautomeric form** |
| 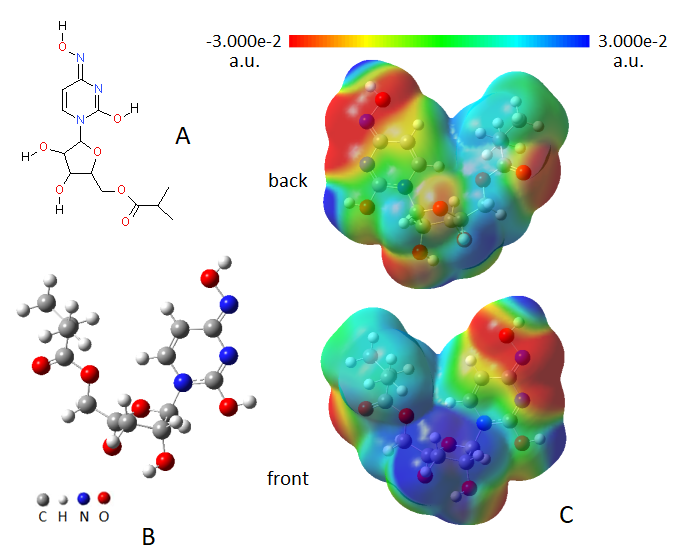  IV |
| **Figure S5-I.** **A)** Structure, **B)** optimized structure {E_(URB3LYP)_= -1197.582307 Hartree (-32587.900713 eV)} and **C)** electrostatic potential (ESP) map (a.u., atomic unit) of molnupiravir. **II –** **A)** HOMO-LUMO energy diagram (E_HOMO_= -6.680 eV, E_LUMO_= -1.570 eV, Δ(E_LUMO_-E_HOMO_) = 5.11 eV), **B)** HOMO-LUMO molecule orbitals and **C)** fragments contributions of HOMO-LUMO orbitals (HOMO = O 27%, N 30%, CH 43% ; LUMO = O 1%, N 28%, CH 71%). **III –** **A)** Structure, **B)** optimized structure {E_(URB3LYP)_= -1197.590564 Hartree (-32588.125397 eV)} and **C)** electrostatic potential (ESP) map (a.u., atomic unit) of keto-oxime tautomer of molnupiravir. **IV –** **A)** Structure, **B)** optimized structure {E_(URB3LYP)_= -1197.559984 Hartree (-32587.293272 eV)} and **C)** electrostatic potential (ESP) map (a.u., atomic unit) of hydroxyl-oxime tautomer of molnupiravir. |
| **3-Thienyl boronic acid (3-TBA)** |
| 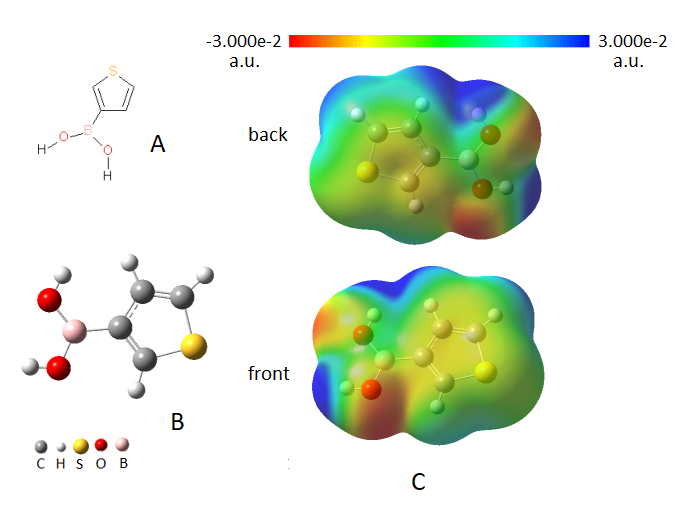  I |
| 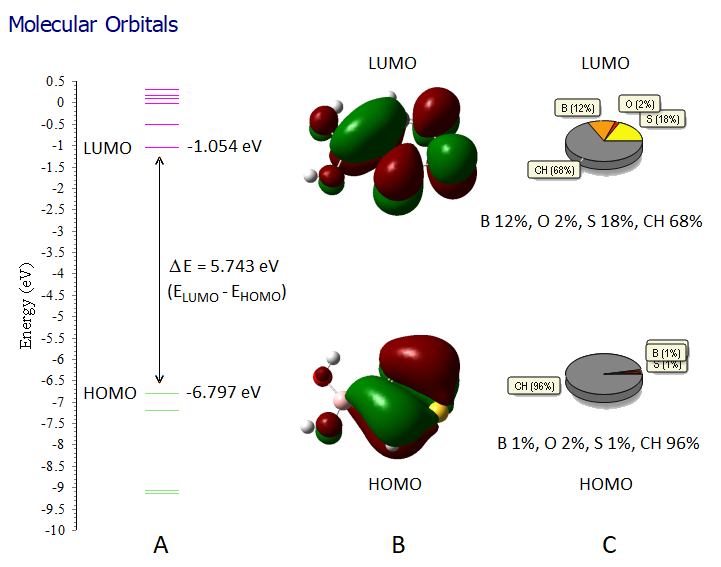  II |
| **Figure S6-I. A)** Structure, **B)** optimized structure {E(RB3LYP)= -729.019940 Hartree (-19837.658993 eV)} and **C)** electrostatic potential (ESP) map (a.u., atomic unit) of 3-TBA. **II–** **A)** HOMO-LUMO energy diagram (E_HOMO_=-6.797 eV, E_LUMO_=-1.054 eV, Δ(E_LUMO_-E_HOMO_) = 5.743 eV), **B)** HOMO-LUMO molecular orbitals and **C)** fragments contributions of HOMO-LUMO orbitals. |

| **Guanine methacrylate (GuaM)** |  |
| --- | --- |
| 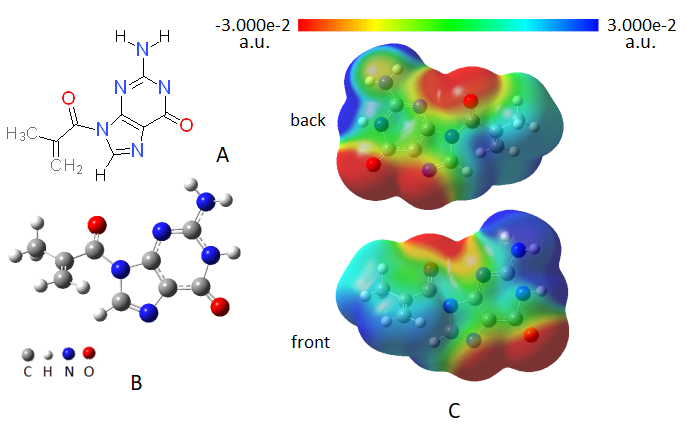  I |  |
| 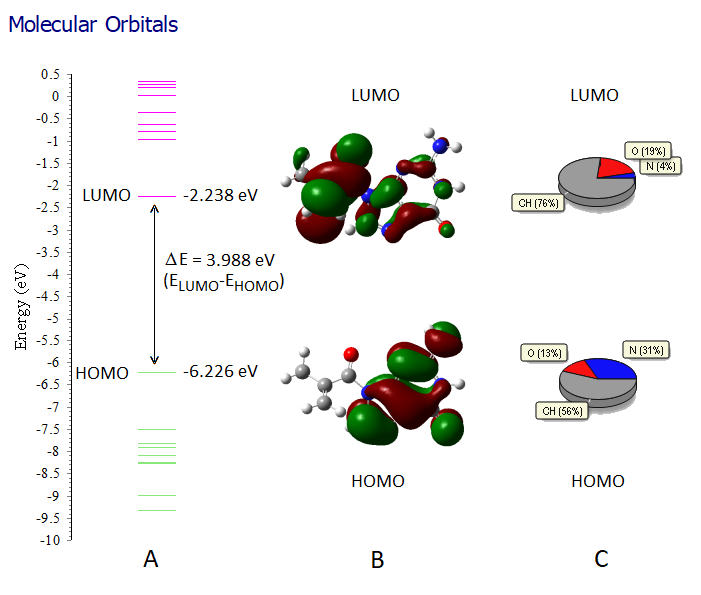  II |  |
| **Figure S7-I. A)** Structure, **B)** optimized structure {E(RB3LYP)= -772.590771 Hartree (-21023.28265 eV)} and **C)** electrostatic potential (ESP) map (a.u., atomic unit) of GuaM. **II –** **A)** HOMO-LUMO energy diagram (E_HOMO_=-6.226 eV, E_LUMO_=-2.238 eV, Δ(E_LUMO_-E_HOMO_) = 3.988 eV), **B)** HOMO-LUMO molecular orbitals and **C)** fragments contributions of HOMO-LUMO orbitals.  **Table S1** – Calculated some electronic, energetic, and geometric global descriptors (QSAR) of compunds using Gaussian 2016 and Discovery Studio 2022 programs.  ***Gaussian 2016***   \| **Global**  **Descriptors, eV** \| **MOL** \| **3-TBA** \| **GuM** \| \| --- \| --- \| --- \| --- \| \| **E_HOMO_** \| -6.680 \| -6.797 \| -6.226 \| \| **E_LUMO_** \| -1.570 \| -1.054 \| -2.238 \| \| **ΔE** \| 5.11 \| 5.743 \| 3.988 \| \| **EA** \| 1.57 \| 1.054 \| 2.238 \| \| **IP** \| 6.68 \| 6.797 \| 6.226 \| \| **χ** \| 4.125 \| 3.926 \| 4.232 \| \| **μ** \| -4.125 \| -3.926 \| -4.232 \| \| **η** \| 2.555 \| 2.872 \| 1.994 \| \| **σ** \| 0.391 \| 0.348 \| 0.502 \| \| **ω** \| 3.33 \| 2.683 \| 4.492 \| \| **ΔN_max_** \| 1.614 \| 1.367 \| 2.122 \| \| **ω+** \| 1.587 \| 1.079 \| 2.624 \| \| **ω-** \| 5.712 \| 5.005 \| 6.856 \|   ΔE=E_LUMO_-E_HOMO_, EA electron affinity, IP ionization potantial, EA = -E_LUMO_, IP = -E_HOMO_ ; χ = absolute electronegativity, μ= chemical potential, η = absolute hardness, σ = softness, ω = electrophilicity index, ΔN_max_ = additional electronic charge from the environment, ω+ = electro-accepting ability, ω- = electro-donating ability. The Py values were calculated in Ref [2]  ***Discovery Studio 2022***   \| **Descriptor** \| **MOL** \| **Keto-Oxime** \| **Hydroxy-Oxime** \| **3-TBA** \| **GuaM** \| \| --- \| --- \| --- \| --- \| --- \| --- \| \| **Molecule Mass,**  **gmol^-1^** \| 329.306 \| 329.306 \| 329.036 \| 127.957 \| 219.076 \| \| **Number of Rotatible Bonds** \| 6 \| 5 \| 5 \| 1 \| 1 \| \| **Number of H- Acceptor** \| 8 \| 8 \| 9 \| 2 \| 4 \| \| **Number of H- Donor** \| 4 \| 2 \| 4 \| 2 \| 2 \| \| **Molecular Polar SASA^$^, Å^2^** \| 208.299 \| 208.299 \| 211.064 \| 130.469 \| 175.99 \| \| **Molecular SASA, Å^2^** \| 503.115 \| 503.115 \| 507.582 \| 287.337 \| 391.599 \| \| **Molecular Polar Surface Area, Å^2^** \| 140.91 \| 140.92 \| 144.41 \| 68.7 \| 102.36 \| \| **Molecular Surface Area**^$$^**, Å^2^** \| 325.59 \| 325.07 \| 328.43 \| 136.27 \| 227.2 \| \| **AlogP^#^** \| -2.745 \| -0.875 \| -0.193 \| 1.445 \| 0.193 \| \| **Molecular Solubility** \| -1.629 \| -1.517 \| -1.664 \| -1.002 \| -1.925 \|   ^$^SASA: solvent accessible surface area; ^$^SA: surface area. The Py values were calculated in Ref [2]. | |
| **Molnupiravir- 3-Thienyl boronic acid -Pyrrole** |  |
|  |  |
| **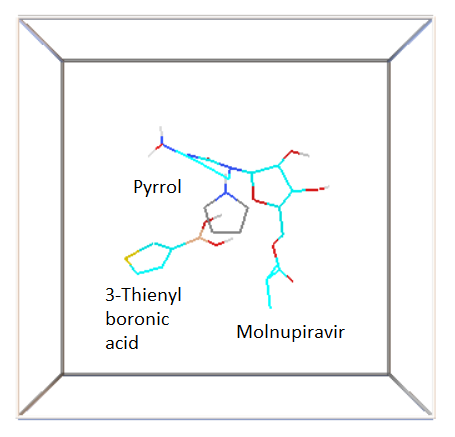**  **Figure S8.** MOL, 3-TBA and Py in box. Coordinates and Box dimensions respectively (Å): center; x= -0.30, y= -0.181, z= 0.789; size; x= 30, y= 29, z= 24. Box dimensions: 30x29x24 Å. Gasteiger force field was used in the calculation. |  |

**Molnupiravir- Guanine methacrylate**

| 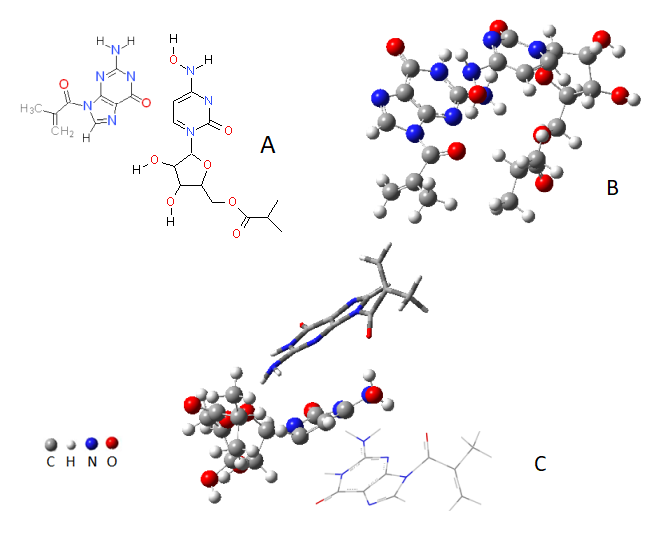 |
| --- |
| **Figure S9. A)** Structure, **B)** optimized structure of MOL-GuaM (1:1) {E_(RB3LYP)_= -1971.388051 Hartree (-53644.244489 eV)} and **C)** optimized structure of molnupiravir-guanine methacrylate (1:2) {E_(RB3LYP)_= -2744.000884 Hartree (-74668.127477 eV)}.  **Table S2.** Elemental values of the EIS measurements' electrical equivalent circuit   \|  \| **EP** \| \| \| **PP** \| \| \| \| --- \| --- \| --- \| --- \| --- \| --- \| --- \| \|  \| **R_p_ (**Ω) \| **R_ct_ (**Ω) \| **CPE** \| **R_p_ (**Ω) \| **R_ct_ (**Ω) \| **CPE** \| \| **Bare GCE** \| 110.0 \| 102 \| 0.872 \| 102.0 \| 93.8 \| 0.867 \| \| **After polymerization** \| 105.0 \| 22000 \| 0.877 \| 101.0 \| 7510 \| 0.877 \| \| **After removal** \| 103.0 \| 825 \| 0.917 \| 90.7 \| 517 \| 0.909 \| \| **After rebinding** \| 99.2 \| 1740 \| 0.890 \| 90.6 \| 1160 \| 0.869 \| |

### **S2.4. Comparison with other methods**

**Table S3** provides a comprehensive overview of the various analytical techniques applied regarding the analytical performance of MOL. When the literature was examined in detail, the excessive use of organic components, long pre-treatment steps, long analysis-time, not being environmental-friendly, and the use of expensive and toxic materials are the disadvantages of spectroscopic and chromatographic techniques. Poly(Py-co-3-TBA)/MOL@MIP/GCE and GuaM/MOL@MIP/GCE sensors have emerged as highly sensitive and selective techniques for the analysis of real samples, surpassing standard methodologies. The experimental results obtained from these sensors demonstrate the superiority of the current method in terms of simplicity, environmental and user-friendliness, cost-effectiveness, and practicality. In conclusion, the findings of the study clearly demonstrate the favorable characteristics of the novel method compared to other methods. The good linearity, reproducibility, low detection limits, selectivity, and stability observed in this study make the novel method a promising choice for accurate and reliable analysis in various fields.

**Table S3.** Summary of selected analytical methods developed for MOL

| **Method** | | **Linear Range** | | **LOD** | **Sample** | **Recovery (%)** | **Ref** |
| --- | --- | --- | --- | --- | --- | --- | --- |
| RP-HPLC | 0.1–60.0 μg/mL | | 0.05 μg/mL | | NA | NA | [7] |
| RP-HPLC-UV | 0.2–80 μg/mL | | 0.04 μg/mL | | Capsule | 100.58 | [8] |
| UHPLC‒MS/MS | 2.5-10 ng/mL | | NA | | Blood  Fetus  Placenta  Amniotic fluid | 97.72-99.4  97.64-101.03  98.13-100.10  99.27-100.87 | [9] |
| UV-Spectroscopic method | 5–25 μg/mL | | 0.02 μg/mL | | Capsule | 99.99 ± 1.23 | [10] |
| HP-TLC | 3.75–100.00 μg/mL | | 1.21 μg/mL | | NA | NA | [11] |
| LC-MS-MS | 2.5–5000 ng/mL | | NA | | Plasma  Saliva | 95-100  65-86 | [12] |
| LC-MS-MS | 20.0–10000.0 ng/mL | | NA | | Plasma | 96.4-97.6 | [13] |
| UPLC-MS-MS | 2.25–100 μg/mL | | 0.68 | | Biological sample | 99.10-100.25 | [14] |
| LC-MS-MS | 1–5000 ng/mL | | NA | | Plasma | 72.2-76.2 | [15] |
| UV | 1–12 μg/mL | | NA | | Capsule | 99.68 | [16] |
| Fluorescence/  MOF method | 10 μM-1 nM | | 2.24 nM | | Capsule  Serum  Urine | 98-102  98.8  99.0 | [17] |
| SWV/rGO/GCE | 0.09-4.57 μM | | 0.03 μM | | Capsule | 99.1 | [18] |
| DPV/Fe_3_O_4_@CPE | 0.25-750 μM | | 0.05 μM | | Tablet | 98.2-100.6 | [19] |
| Poly(Py-co-3-TBA)/MOL@MIP/GCE | 7.5-250 pM | | | 0.60 pM | Capsule  Serum | 101.35  99.09 | **This study** |
| GuaM/MOL@MIP/GCE | 0.75-25 pM | | | 0.13 pM | Capsule  Serum | 99.40  100.97 | **This study** |

NA: Not available, DPV: Differential pulse voltammetry, SWV: Square-wave voltammetry, HPLC: High-performance liquid chromatography, LC: Liquid chromatography, TLC: Thin layer chromatography, MS/MS: Tandem mass spectrometry, GCE: Glassy carbon electrode, CPE: Carbon paste electrode, MOF: Metal–organic frameworks

### **S3. Structural characterization**





**Figure S10.** FTIR spectrum of molnupiravir





**Figure S11.** FTIR spectrum of pyrrole monomer





**Figure S12.** FTIR spectrum of 3-TBA monomer





**Figure S13.** FTIR spectrum of GuaM monomer

**S4.** Synthesis of functional monomer, GuaM

1H NMR spectrum of GuaM was used to demonstrate the characteristic peaks from the groups in the monomer. Peaks in the 1H-NMR spectrum were observed as (1H, 400 MHz, DMSO- d6); In this analysis, NH_2_ protons are seen between 4.5-5.0. The peaks observed at 5.56 and 5.25 are due to olefinic protons. From these data, it can be said that the synthesis process was carried out successfully.

**
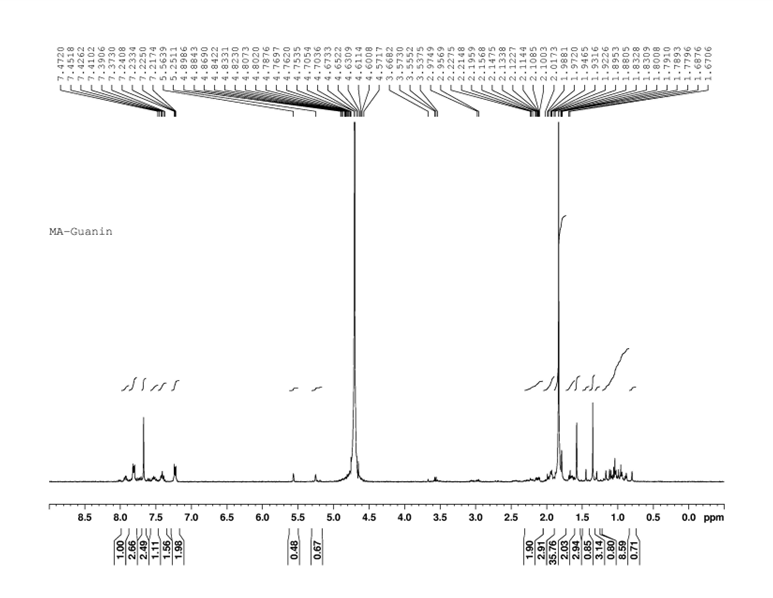
**

**Figure S14.** ^1^H NMR spectrum of GuAM monomer.

**References**

1. Armutcu C (2020) Rapid and Specific Purification of RNA by PolyUracil Membranes. Adiyaman University Journal of Science 10:466–482. <https://doi.org/10.37094/adyujsci.780049>

2. M.J. Frisch, G.W. Trucks, H.B. Schlegel, G.E. Scuseria, M.A. Robb, J.R. Cheeseman, et al., Gaussian 2016 Revision C.01, Gaussian Inc, Wallingford CT (2009). R.D. Dennington II, T.A. Keith, J.M. Millam, GaussView. Ver. 6.1.1, Semichem, Inc., Wallingford CT, (2009).

3. S. Leonid, Chemissian. Ver. 4.38, Available from: http://www.chemissian.com.

(Accessed February 2023).

4. Dassault Systèmes BIOVIA, Discovery Studio Modeling Environment, Release 22.1.0.2197, San Diego: Dassault Systèmes, 2021.

5 A) M.G. Morris, R. Huey, W. Lindstrom, M. F. Sanner, R. K. Belew, D. S. Goodsell, and A. J. Olson (2009). “Autodock4 and AutoDockTools4: automated docking with selective receptor flexiblity”, J. Computational Chemistry 2009, 16: 2785-91. B) J. Eberhardt, D. Santos-Martins, A. F. Tillack, and S. Forli. (2021). “AutoDock Vina 1.2.0: New Docking Methods, Expanded Force Field, and Python Bindings”, Journal of Chemical Information and Modeling”, J. Chem. Inf. Model. 61, 8, 3891–3898.C) O. Trott, A. J. Olson, (2010). “AutoDock Vina: improving the speed and accuracy of docking with a new scoring function, efficient optimization and multithreading”, Journal of Computational Chemistry 31, 455-461.

6. Cetinkaya A, Unal MA, Nazır H, et al (2023) Two different molecularly imprinted polymeric coating techniques for creating sensitive and selective electrochemical sensors for the detection of Ribavirin. Sens Actuators B Chem 389:. https://doi.org/10.1016/j.snb.2023.133914

7. Reçber T, Timur SS, Erdoğan Kablan S, et al (2022) A stability indicating RP-HPLC method for determination of the COVID-19 drug molnupiravir applied using nanoformulations in permeability studies. J Pharm Biomed Anal 214:. https://doi.org/10.1016/j.jpba.2022.114693

8. Annadi AM, El Zahar NM, El-Din A. Abdel-Sattar N, et al (2022) Development and validation of molnupiravir assessment in bulk powder and pharmaceutical formulation by the RP-HPLC-UV method. RSC Adv 12:34512–34519. https://doi.org/10.1039/d2ra05066h

9. Chang C-H, Peng W-Y, Lee W-H, et al (2023) Transfer and biotransformation of the COVID-19 prodrug molnupiravir and its metabolite β-D-N4-hydroxycytidine across the blood-placenta barrier

10. Sharaf YA, El Deeb S, Ibrahim AE, et al (2022) Two Green Micellar HPLC and Mathematically Assisted UV Spectroscopic Methods for the Simultaneous Determination of Molnupiravir and Favipiravir as a Novel Combined COVID-19 Antiviral Regimen. Molecules 27:. https://doi.org/10.3390/molecules27072330

11. Saraya RE, Deeb S El, Salman BI, Ibrahim AE (2022) Highly sensitive high-performance thin-layer chromatography method for the simultaneous determination of molnupiravir, favipiravir, and ritonavir in pure forms and pharmaceutical formulations. J Sep Sci 45:2582–2590. https://doi.org/10.1002/jssc.202200178

12. Amara A, Penchala SD, Else L, et al (2021) The development and validation of a novel LC-MS/MS method for the simultaneous quantification of Molnupiravir and its metabolite ß-d-N4-hydroxycytidine in human plasma and saliva. J Pharm Biomed Anal 206:. https://doi.org/10.1016/j.jpba.2021.114356

13. Gouda AS, Marzouk HM, Rezk MR, et al (2022) A validated LC-MS/MS method for determination of antiviral prodrug molnupiravir in human plasma and its application for a pharmacokinetic modeling study in healthy Egyptian volunteers. J Chromatogr B Analyt Technol Biomed Life Sci 1206:. https://doi.org/10.1016/j.jchromb.2022.123363

14. Nakka S, Muchakayala SK, Manabolu Surya SB (2023) A sensitive UPLC-MS/MS method for the simultaneous assay and trace level genotoxic impurities quantification of SARS-CoV-2 inhibitor-Molnupiravir in its pure and formulation dosage forms using fractional factorial design. Results Chem 6:101019. https://doi.org/10.1016/j.rechem.2023.101019

15. Parsons TL, Kryszak LA, Marzinke MA (2021) Development and validation of assays for the quantification of β-D-N4-hydroxycytidine in human plasma and β-D-N4-hydroxycytidine-triphosphate in peripheral blood mononuclear cell lysates. J Chromatogr B Analyt Technol Biomed Life Sci 1182:. https://doi.org/10.1016/j.jchromb.2021.122921

16. Abdelazim AH, Abourehab MAS, Abd Elhalim LM, et al (2023) Green adherent spectrophotometric determination of molnupiravir based on computational calculations; application to a recently FDA-approved pharmaceutical dosage form. Spectrochim Acta A Mol Biomol Spectrosc 285:. https://doi.org/10.1016/j.saa.2022.121911

17. Younis HM, Youssef AO, El-Sheikh SM, et al (2023) Preparation and Characterization of Fe-Gallic acid MOF for determination of antiviral Molnupiravir as inhibitor for RNA Corona virus replication. Microchemical Journal 194:109297. https://doi.org/10.1016/j.microc.2023.109297

18. Kablan SE, Reçber T, Tezel G, et al (2022) Voltammetric sensor for COVID-19 drug Molnupiravir on modified glassy carbon electrode with electrochemically reduced graphene oxide. Journal of Electroanalytical Chemistry 920:. https://doi.org/10.1016/j.jelechem.2022.116579

19. Vural K, Karakaya S, Dilgin DG, et al (2023) Voltammetric determination of Molnupiravir used in treatment of the COVID-19 at magnetite nanoparticle modified carbon paste electrode. Microchemical Journal 184:. https://doi.org/10.1016/j.microc.2022.108195
